# Bone mineral density and carotid atherosclerosis in systemic lupus erythematosus: a controlled cross-sectional study

**DOI:** 10.1186/s13075-015-0595-4

**Published:** 2015-03-25

**Authors:** Sofia Ajeganova, Thomas Gustafsson, Tomas Jogestrand, Johan Frostegård, Ingiäld Hafström

**Affiliations:** Department of Medicine, Unit of Rheumatology, Karolinska Institutet at Karolinska University Hospital Huddinge, Stockholm, 141 86 Sweden; Department of Clinical Physiology, Karolinska Institutet at Karolinska University Hospital Huddinge, Stockholm, 141 86 Sweden; Section of Immunology and Chronic disease, Institute of Environmental Medicine, Karolinska Institutet, Stockholm, 171 77 Sweden

## Abstract

**Introduction:**

As osteoporosis is reported to be associated with atherosclerosis in the general population we examined the relationship between bone mass and carotid measurements in patients with systemic lupus erythematosus (SLE) and controls, and possible links between them in SLE.

**Methods:**

In a cross-sectional study, 111 SLE-patient were compared with 111 age- and sex-matched controls, mean age 48.7(12.9) years, 89% were women, of which 51% postmenopausal. Carotid intima media thickness (cIMT), carotid plaque occurrence and echogenicity were determined by B-mode ultrasound and bone mineral density (BMD) by dual-energy X-ray absorptiometry (DXA).

**Results:**

BMD and cIMT were inversely associated both in patients and controls. Patients, but not controls, with carotid plaque had higher cIMT at low BMD than at normal BMD, p = 0.010. Logistic regression indicated more than doubled odds ratio (OR) of carotid plaque in patients, particularly in post-menopausal women, than in controls in relation to all BMD measurements. For low BMD at hip, significant increased OR for echolucent plaque was shown for patients compared with controls.

In patients, significant impact of age, body mass index, smoking, systolic blood pressure, blood lipids, diabetes mellitus, impaired renal function, low levels of complement C3 and C4, history of nephritis, SLE-damage index and ever use of antimalarial was found for association between BMD and higher cIMT and carotid plaque. In multivariate regression, low C4 was independent contributor to association between total BMD and upper cIMT tertile, accounted for OR (95% confidence interval) of 3.2 (1.03-10.01), and also for association with bilateral carotid plaque, OR of 4.8 (1.03-22.66). The contribution of low C4 for the association between BMD and carotid atherosclerosis was enhanced within the second and third tertiles of total BMD.

**Conclusion:**

This study is the first to demonstrate inverse association between BMD and carotid measurements in both SLE-patients and controls. Our results suggest that SLE-patients may suffer higher burden of (sub)clinical atherosclerotic disease, especially presence of both echolucent and echogenic plaque, than controls with the same bone mineral status. Low complement C4 seems to play an important role in earlier development of carotid atherosclerosis already within (sub)normal ranges of total BMD in patients.

**Electronic supplementary material:**

The online version of this article (doi:10.1186/s13075-015-0595-4) contains supplementary material, which is available to authorized users.

## Introduction

Osteoporosis and osteopenia have been reported to be associated with atherosclerosis and cardiovascular disease (CVD) in the general population [1-5]. Both osteoporosis and atherosclerosis are common disorders of ageing and are among the main causes of morbidity and mortality in elderly [1,6]. Low bone mineral density (BMD) has also been associated with severity of carotid atherosclerosis and increased risk of echogenic calcified atherosclerotic plaque [7-9].

Whether these two clinical conditions, also defined as systemic disorders, are linked by common pathogenic mechanisms or atherosclerosis per se*,* or whether osteoporosis is a cardiovascular risk factor remains unclear. Risk factors for CVD, such as as hypertension, smoking, dyslipidaemia, diabetes and oxidative stress have also been associated with increased risk of low BMD [10,11]. Epidemiologic studies have consistently reported inverse associations between BMD and structural and functional measures of atherosclerosis, seemingly independent of age, sex and shared traditional cardiovascular risk factors [9,12,13].

Osteoporosis and atherosclerosis are common clinical problems in inflammatory diseases, and inflammation seems to play an important role in both conditions [14]. In systemic lupus erythematosus (SLE), cross-sectional studies have revealed several disease-related risk factors for low BMD, that is, long disease-duration, elevated markers of inflammation, impaired renal function, chronic organ damage and cumulative glucocorticoid dose [15-19]. To date, underlying inflammatory immunological pathways for atherosclerosis and an increased risk of CVD in SLE have been recognized. Disease duration, disease activity, damage indices and higher glucocorticoid dose have proved the most reproducible non-traditional risk factors for progression of atherosclerosis and CVD [20-23]. Hence, it certainly seems plausible to think that there is also a relationship between BMD and atherosclerosis in SLE, but the evidence is lacking [24].

To our knowledge, no previous studies have evaluated whether or not the association between BMD and atherosclerosis differs between patients with SLE and individuals in the general population. Here, we examined the association between bone mass and carotid measurements in patients with SLE and age- and sex-matched population-based controls. Then, we aimed to analyze if disease-related factors, aside of traditional risk factors, may explain the link between bone mineral health and carotid atherosclerosis in SLE.

## Methods

### Patients

This study was based on participants included in the SLE vascular impact cohort study (SLEVIC), details of which have been published previously [25]. In brief, 114 patients with SLE, who fulfilled the 1982 revised criteria of the American College of Rheumatology (ACR) for SLE [26] and were younger than 70 years, and 122 aged- and sex-matched controls (recruited randomly from the same catchment area), were enrolled into the SLEVIC cohort.

For the current analysis, participants of the derivation cohort who underwent both dual-energy x-ray absorptiometry (DXA) and carotid ultrasound examination were identified. We then confirmed that each patient and his/her control were similar in age and sex; these characteristics are not of interest in themselves in the current study, but are known to be estimations of the variables we intended to study, that is, BMD and carotid measures. Thus, in this study we assessed a cohort of 111 patients and 111 controls who were matched individually. The study was approved by the Regional Ethic committee at Karolinska Institutet, Stockholm, and was performed in accordance with the Declaration of Helsinki. All study participants provided written informed consent before entering the study.

### Clinical assessment

Body mass index (BMI) was calculated as weight/height^2^ (kg/m^2^). Blood pressure was recorded in a seated position with an automatic device by a trained nurse after 5 minutes rest. Hypertension was defined as systolic blood pressure ≥140 mmHg, diastolic blood pressure ≥90 mmHg and/or the use of antihypertensive medications and/or previous diagnosis by a physician. Participants were considered ever-smokers if they smoked at inclusion or had smoked before. We recorded personal history of CVD (coronary or peripheral arterial disease, ischaemic stroke, transient ischaemic attack) and medication for all participants using a questionnaire, and also medical record review for the patients. Cumulative and average dose of glucocorticoids (GC) and duration of GC therapy were calculated. SLE activity was assessed with the systemic lupus erythematosus diseases activity index (SLEDAI) [27], and organ damage was measured using the Systemic Lupus International Collaborating Clinics (SLICC) damage index [28].

### Laboratory assessment

Blood samples were collected after an overnight fast. The biochemical analyses were determined by standard laboratory methods. Hypercholesterolaemia was determined by fasting plasma cholesterol ≥5.0 mmol/l and/or the use of lipid-lowering medications, and diabetes mellitus was determined by fasting blood glucose ≥7.0 mmol/l and/or the use of anti-diabetic medications. Hypercholesterolaemia and diabetes were also considered present if diagnosed by a physician.

### Carotid ultrasound

As described in detail previously [25], the right and left carotid arteries were examined with a duplex scanner (Sequoia, Siemens Acuson, Mountain View, CA, USA) using a -MHz linear array transducer. The far wall of the common carotid artery (CCA), 0.5 to 1.0 cm proximal to the beginning of the carotid bulb, was used for measurements of the carotid intima-media thickness (cIMT). The cIMT was defined as the distance between the leading edge of the lumen-intima echo and the leading edge of the media-adventitia echo. The examinations were digitally stored for subsequent analyses by a computer system [29] with automated tracing of echo interfaces and measurements of distances between the wall echoes within a 10-mm-long section of CCA in late diastole, defined by a simultaneous electrocardiographic recording. The mean values of the cIMT within the 10-mm-long section were calculated. The mean cIMT was calculated as (right + left)/2. When a plaque was observed in the region of the CCA measurements, the cIMT was not measured.

Carotid plaque was defined as a localized intima-media thickening >1 mm and at least 100% increase in thickness compared with adjacent wall segments. Plaque was screened for in the common, internal and external carotid arteries and described as the absence of plaque, the presence of unilateral plaque, or bilateral plaque. Plaque morphology in terms of echogenicity was assessed in a modified version of the classification proposed by Gray-Weale *et al*. [30] and graded from 1 to 4 ranging from low to strong echogenicity. Echolucency was defined with the arterial lumen as reference and echogenicity with the far wall adventitia as reference. We considered grade-1 and/or grade-2 plaques to be echolucent, and echogenic plaque was denoted as grade 3 and/or grade 4.

The difference between repeated measurements of cIMT was 4.9% (coefficient of variation) using the automated analysis system. Repeated classification of plaque morphology showed a correlation coefficient of 0.7 (*P* <0.05) between the first and second classification (n = 50).

### Bone mineral density

BMD was measured by DXA with a Lunar densitometer (Lunar Corporation, Expert-XL software version 1.7, Madison, USA, 1998). All BMD measurements were expressed in absolute values in gram of bone mineral per square centimetre (g/cm^2^) and as *T*-score, that is, the number of SDs above or below the mean results of healthy, young sex-matched adults (values obtained from the Lunar combined European/US reference population). Results are given for total body BMD, BMD at the hip (total hip and femoral neck) and at the lumbar spine (vertebrae L1 to L4; antero-posterior view). Low BMD was defined as a *T*-score below −1.0, that is, osteopenia (*T*-score −1.0 to −2.5) and osteoporosis (*T*-score below −2.5) according to the World Health Organisation (WHO) criteria for osteoporosis [31].

### Statistical analysis

Continuous variables were summarized as mean (SD) or median (IQR), as appropriate, and categorical variables as frequencies (percentages). The chi-square test or Fisher's exact test, or one-way analysis of variance (ANOVA) was used for comparison as appropriate. Pearson correlation was used to determine the bivariate association between BMD and cIMT in the study groups separately. Taking into account the possibility of non-linear continuous variables, such as cIMT and BMD, we stratified these variables into tertiles.

Regression models were used to test independent association between BMD and carotid measurements in the patients and controls (cIMT, presence or absence of carotid plaque as the dependent variables, and BMD and study group as the independent variables), and to identify factors contributing to the association with carotid atherosclerosis in the patients (traditional risk factors and disease-related factors as the independent variables). For the participants with plaque, the association between BMD and plaque echogenicity was also tested by logistic regression, where plaque echogenicity (graded in categories) was the dependent variable, and BMD and study group were independent variables. To determine which factors had significant influence on the relationship between BMD and carotid outcomes, we performed stepwise multivariate analyses. The possible confounding variables for the first-step models, such as hypertension, hypercholesterolaemia, diabetes mellitus, history of CVD, BMI and smoking, were all a priori prespecified (as well-known risk factors for atherosclerosis) and chosen for final models by Forward Wald selection procedure. The second-step model selected significant disease-related factors by the same procedure. To the final model variables identified in the first steps were entered together. Variables of blood lipids were added to the model individually. The groups were matched by sex and age, thus, these covariates were not included in the main analysis. Results of the logistic regression models are presented as odds ratio (OR) with corresponding 95% CI, excluding zero which indicated significant differences. The ability of BMD and disease-related covariates to explain carotid atherosclerosis in the patient group was assessed by receiver-operating characteristic (ROC) curves. Two tailed significance tests at *P*-values <0.05 were considered to be statistically significant. IBM SPSS software version 22 (SPSS Inc., Chicago, IL, USA) was used for the computations.

## Results

### Characteristics of patients and controls

Data on the whole original SLEVIC study population have been presented previously [25]. The demographic and clinical characteristics of patients and controls assembled in the current analysis are shown in Table 1. Thus, significant differences between the groups were found with respect to presence of hypertension, history of previous CVD, and to higher high-sensitivity C-reactive protein (hsCRP) and ESR measures in the patients.

**Table 1:** **Demographic, clinical and carotid ultrasound characteristics of patients with systemic lupus erythematosus and sex- and age-matched controls**

	**SLE, n = 111**	**Controls, n = 111**	***P*** **-value**
Age, years	48.6 (13.0)	48.8 (12.9)	0.939
Females, n %	99 (89.2%)	99 (89.2%)	1.0
Postmenopausal, n (%)	48 (48.5%)	52 (52.5%)	0.572
Body mass index, kg/m^2^	24.9 (4.4)	25.1 (4.4)	0.657
Ever smoking, n (%)	62 (55.9%)	61 (55.0%)	0.893
Hypertension, n (%)	65 (58.6%)	26 (23.4%)	<0.001
Hypercholesterolaemia, n (%)	54 (48.6%)	55 (49.5)	0.893
Diabetes, n (%)	6 (5.4%)	3 (2.7%)	0.499
History of cardiovascular disease, n (%)	15 (13.5%)	2 (1.8%)	0.001
High-sensitivity C-reactive protein, mg/l	2.0 (0.8 to 4.8)	1.2 (0.5 to 2.5)	0.001
Erythrocyte sedimentation rate, mm/h	21.0 (12.3 to 31)	8.0 (5.0 to 13.0)	<0.001
**Carotid measurements**			
Intima media thickness, mean, mm	0.62 (0.13)	0.63 (0.13)	0.766
Plaque presence, n (%)			0.023
No plaque	61 (55.0%)	80 (72.1%)	
Unilateral	20 (18.0%)	15 (13.5%)	
Bilateral	30 (27.0%)	16 (14.4%)	
Plaque echogenicity, n (%)			
Echolucent (versus no plaque)	42 (37.8%)	28 (25.2%)	0.022
Echogenic (versus no plaque)	13 (11.7%)	5 (4.5%)	0.020

Values are number (percent), mean (SD) or median (IQR). *P*-values indicate between-group differences.

Regarding SLE-disease related features (described in Table 2), the majority of the patients had had long-standing disease at study inclusion, and had mild disease activity and low severity. A third of the patients had a history of lupus nephritis. Low complement levels (C3 <0.67 g/l, C4 <0.13 g/l and C1q <70 mg/l) were found in 10%, 32% and 22% of the patients, respectively. About 90% of the patients had at some time point been on medication with GC, and 85% with antimalarial drugs.

**Table 2:** **Clinical features of the 111 systemic lupus erythematosus (SLE) patients**

**Clinical variables**	**Value**
Duration SLE since diagnosis, years	9.0 (5.0 to 17.0) (range 0 to 40)
Systolic blood pressure, mmHg	127.7 (20.1)
Diastolic blood pressure, mmHg	78.7 (12.1)
A history of lupus nephritis, n (%)	38 (34.2%)
SLEDAI	2.0 (0 to 5.25) (range 0 to 27)
SLICC index	1 (0 to 3) (range 0 to 8)
Complement C3, g/l	0.93 (0.22)
Complement C4, g/l	0.17 (0.12 to 0.23)
Complement C1q, mg/l	88.6 (28.1)
**Treatment variables**	
Current use of immune-modulatory treatment, n (%)	94 (84.7%)
Glucocorticoids	67 (60.4%)
Glucocorticoid dose (mg/day)	5.0 (3.75 to 10.0)
Antimalarial	53 (47.7%)
Azathioprine	24 (21.6%)
Mycophenolatmofetil	9 (8.1%)
Cyclosporine A	7 (6.3%)
Methotrexate	10 (9%)
No disease-modifying anti-rheumatic drugs	17 (15.3%)
Ever use of glucocorticoids, n (%)	99 (89.2%)
Total duration of glucocorticoid use, months	54 (14 to 113)
Total glucocorticoid cumulative dose, g	11.4 (3.5 to 24.5)
Average glucocorticoid dose, mg/day	4.3 (3.4)
Last year glucocorticoid cumulative dose, g	1.4 (0 to 2,3)
Last year glucocorticoid average dose, mg/day	3.8 (0 to 6.3)

Values are number (percent), mean (SD) or median (IQR) when not indicated otherwise. SLEDAI, SLE disease activity index; SLICC, Systemic Lupus International Collaborating Clinics.

### Carotid IMT and plaque presence

Mean cIMT did not differ between the patients with SLE and the controls. On the other hand, the frequency of carotid plaque was higher in the patients than in the controls, 45% versus 28%, *P* = 0.008. In both groups echolucent plaques were more common than echogenic, and both kinds of plaque were more common in the patients than in controls (Table 1).

### Bone mineral density

The patients had significantly lower mean BMD measurements at the total hip and femoral neck compared with the controls; this was observed in the subgroup of postmenopausal women (Table 3). However, there were no differences between patients and controls in total and lumbar spine BMD. Presence of osteoporosis and osteopenia, according to WHO criteria, in the lumbar spine did not differ between the groups. On the other hand, a higher proportion of low BMD in the hip was found in patients compared with controls, 58% versus 45%.

**Table 3:** Bone mineral density (BMD) in all patients with systemic lupus erythematosus (SLE) and sex- and age-matched controls (*subanalysis of postmenopausal women only)

	**SLE, n = 111**	**Controls, n = 111**	***P*** **-value**
**BMD, mg/cm** ^**2**^			
Total body	1.119 (0.127)	1.130 (0.137)	0.563
Postmenopausal women*	1.056 (0.106)	1.064 (0.017)	0.724
Lumbar spine, vertebrae L1 to L4	1.134 (0.120)	1.153 (0.177)	0.456
Postmenopausal women*	1.058 (0.221)	1.075 (0.180)	0.668
Total hip	0.959 (0.151)	0.998 (0.139)	0.046
Postmenopausal women*	0.903 (0.126)	0.949 (0.138)	0.087
Femoral neck	0.909 (0.142)	0.958 (0.162)	0.017
Postmenopausal women*	0.846 (0.110)	0.893 (0.128)	0.048
**T-score:**			
Total body	0.27 (1.20)	0.35 (1.23)	0.620
Postmenopausal women*	−0.23 (1.04)	−0.16 (1.22)	0.754
Lumbar spine, vertebrae L1 to L4	−0.45 (1.38)	−0.38 (1.41)	0.683
Postmenopausal women*	−0.96 (1.46)	−0.97 (1.46)	0.974
Total hip	−0.42 (1.24)	−0.10 (1.09)	0.045
Postmenopausal women*	−0.82 (1.07)	−0.43 (1.15)	0.084
Femoral neck	−0.66 (1.18)	−0.32 (1.07)	0.026
Post-menopausal women*	−1.12 (0.92)	−0.73 (1.06)	0.054
**BMD groups**			
Normal total body BMD	32 (28.8%)	45 (40.5%)	0.067
Postmenopausal women*	6 (12.5%)	11 (21.2%)	0.250
Low BMD, lumbar spine	60 (54.1%)	52 (46.8%)	0.283
Postmenopausal women*	33 (68.8%)	36 (69.2%)	0.959
Low BMD, total hip	64 (57.7%)	50 (45%)	0.060
Postmenopausal women*	35 (72.9%)	33 (63.5%)	0.311
Low BMD, femoral neck	69 (62.2%)	58 (52.3%)	0.136
Postmenopausal women*	38 (79.2%)	37 (73.1%)	0.476
Low BMD, at least one region	79 (71.2%)	66 (59.5%)	0.067
Postmenopausal women*	42 (87.5%)	41 (78.8%)	0.250
**Treatments for osteoporosis,** n (%):			
Calcium supplement and/or vitamin D	71 (64.0%)	7 (6.3%)	<0.001
Postmenopausal women*	34 (70.8%)	6 (11.5%)	<0.001
Bisphosphonates	14 (12.6%)	4 (3.6%)	0.014
Postmenopausal women*	10 (20.8%)	4 (7.7%)	0.058
Hormone replacement therapy	2 (1.8%)	3 (2.7%)	1.0
Postmenopausal women*	2 (4.2%)	3 (5.8%)	1.0

Values are number (percentage and mean (SD)). *P*-values indicate between-group differences. Low BMD is defined as a *T*-score < −1.0 SD.

Two-thirds of the patients were taking treatment for osteoporosis, mostly calcium supplements and vitamin D (64%), bisphosphonates (13%), and also hormone replacement therapy (2%). Only about one tenth of controls were prescribed medication for osteoporosis (Table 3).

### Relation of BMD with carotid IMT measurements

**Figure 1:**
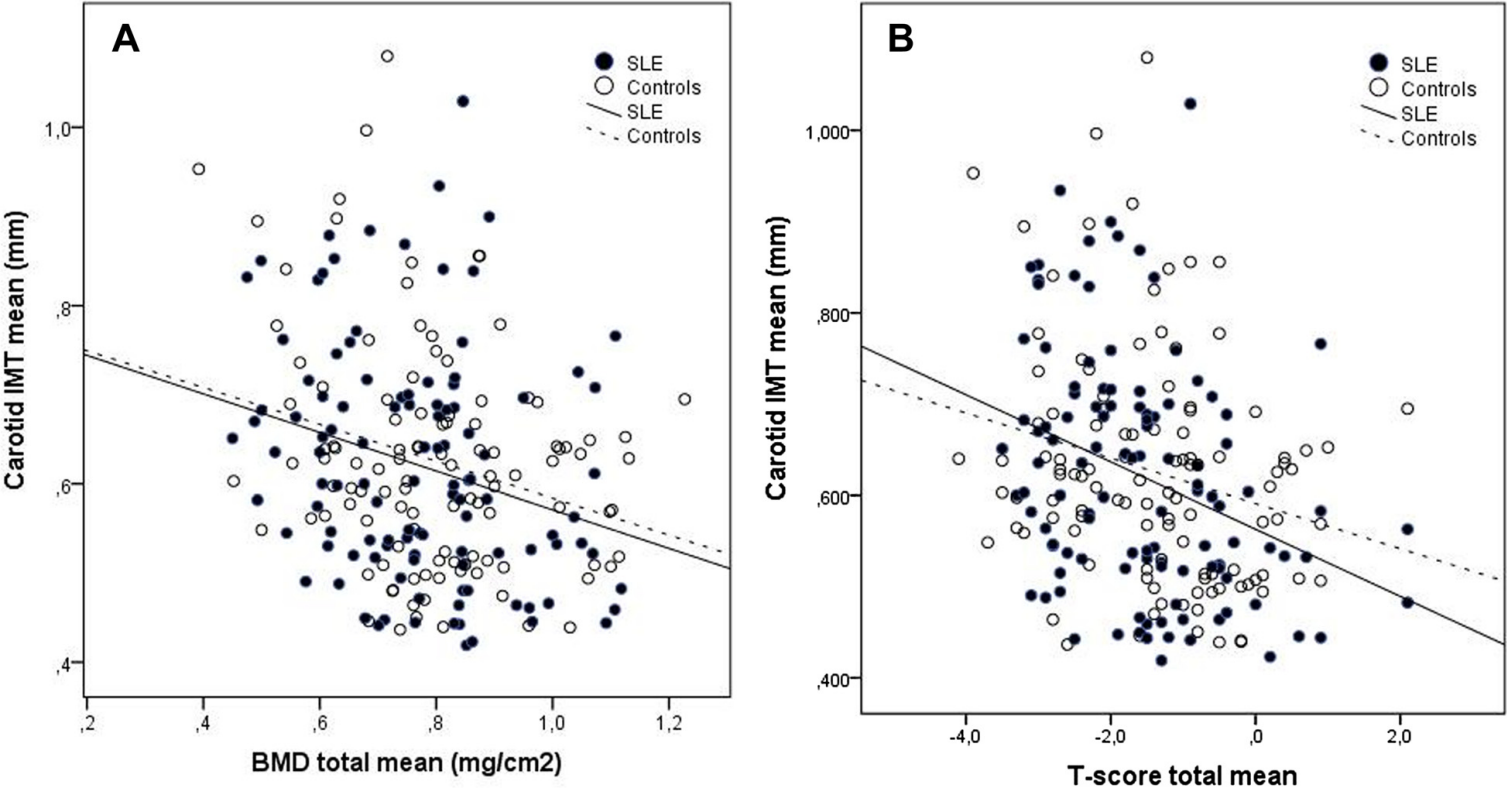
**Scatter plot of the relationship between total body bone mineral density (BMD) (A) and** ***T*** **-score (B), lowest at any region, and carotid intima-media thickness (IMT) measurements in 111 patients with systemic lupus erythematosus (SLE) and 111 sex- and age-matched controls. (A)** The Pearson correlation coefficient was −0.26, *P* = 0.006 for the patients, and −0.27, *P* = 0.005 for the controls. **(B)** The respective coefficients were −0.33, *P* = <0.001, and −0.24, *P* = 0.012.

When stratification of data according to normal BMD and low BMD was done, we found no difference in age, sex or BMI between the study groups, with age of about 42 years in individuals with normal BMD (88% women) and 52 years in those with low BMD values (97% women), and BMI of about 25 kg/m^2^ in all. BMD was negatively associated with cIMT measurements in both patients and controls, as displayed in Figure 1A-B. No statistically significant difference between the groups was observed for this association.

Both patients and controls had higher cIMT if they had low BMD, that is, mean cIMT (mm) 0.64 (0.13) in the patients with low BMD, versus 0.57 (0.12) with normal BMD, *P* = 0.008; and corresponding means cIMT (mm) 0.65 (0.13) versus 0.58 (0.10), *P* = 0.003 in the controls. At low BMD (79 patients and 66 controls), the patients with any carotid plaque had higher cIMT than at normal BMD, respective means cIMT (mm) 0.72 (0.11) and 0.61 (0.15), *P* = 0.010, whereas such a difference was not found in the controls.

At normal BMD (32 patients and 45 controls), echolucent plaques were found at numerically lower cIMT values in the patients than in the controls, respective means cIMT (mm) 0.65 (0.16) versus 0.73 (0.17). Similarly the cIMT values were lower in patients with echogenic carotid plaque than in the controls with that type of plaque at normal BMD, means cIMT 0.67 (0.04) versus 1.08 (0).

### Relation between BMD and occurrence and echogenicity of carotid plaques

**Figure 2:**
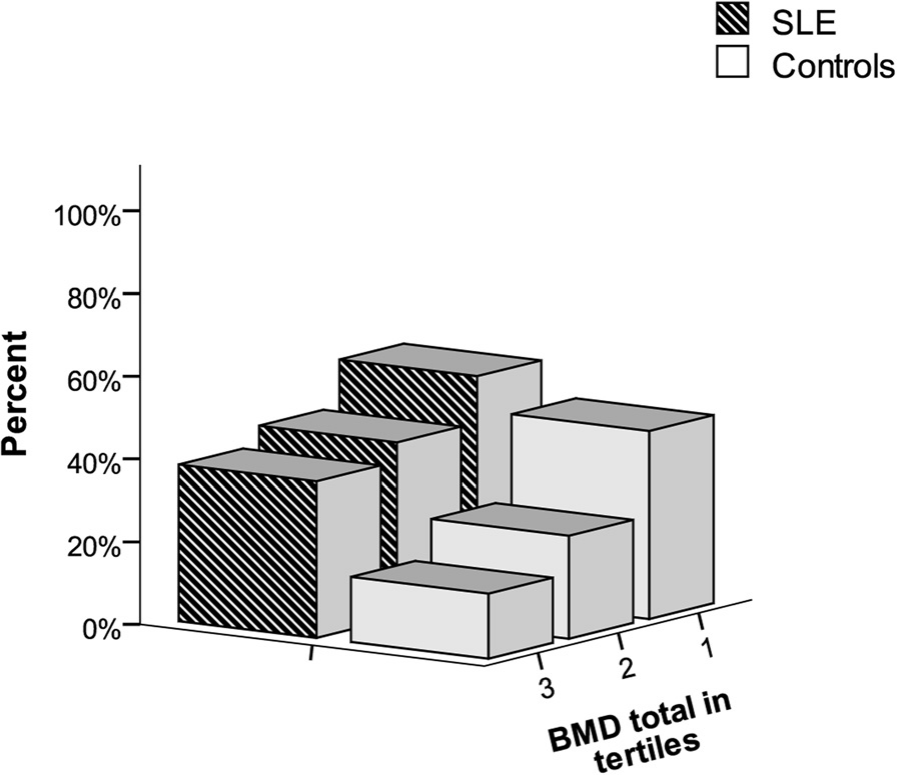
**Distribution of carotid plaque in relation to mean total body bone mineral density (BMD) by tertiles in patients with systemic lupus erythematosus (SLE) and sex- and age-matched controls.** BMD tertiles: 1 <1.067, 3 >1.183 mg/cm^2^.

In both study groups, carotid plaques were more prevalent at lower BMD values (Figure 2). The group difference in the relationship between BMD measurements and occurrence of carotid plaques derived from logistic regression is shown in Table 4. The results display a systematic pattern for more than doubled OR of detection with carotid plaque in the patients than in the controls in relation to all BMD measurements. In multivariate analysis adjusted for hypertension, hypercholesterolaemia and history of CVD, the point estimates were similar, but weaker association was indicated. For postmenopausal women, however, approximately 2.5-times increased OR were observed for carotid plaque across all BMD measurements in the patients compared with the controls. The results hardly changed when CRP, ESR and use of treatment for osteoporosis were included in the regression models.

**Table 4:** **Odds ratio for carotid plaque in relation to bone mineral density (BMD) in systemic lupus erythematosus (SLE) patients compared with matched controls**

	**Unadjusted models**	***P*** **-value**	**Multivariate adjusted models**	***P*** **-value**	Postmenopausal* adjusted models	***P*** **-value**
	**Odds ratio (95% CI)**		**Odds ratio (95% CI)**		**Odds ratio (95% CI)**	
**BMD**						
Total body	2.11 (1.20, 3.72)	0.010	1.77 (0.96, 3.24)	0.066	2.62 (1.10, 6.24)	0.030
Lumbar spine, vertebrae L1 to L4	2.08 (1.18, 3.66)	0.011	1.75 (0.96, 3.21)	0.070	2.59 (1.08, 6.19)	0.032
Total hip	2.00 (1.14, 3.52)	0.016	1.74 (0.95, 3.18)	0.074	2.71 (1.13, 6.52)	0.026
Femoral neck	2.06 (1.17, 3.63)	0.013	1.76 (0.96, 3.22)	0.067	2.72 (1.14, 6.46)	0.024
***T*** **-score**						
Total body	2.12 (1.20, 3.74)	0.010	1.78 (0.97, 3.26)	0.064	2.63 (1.10, 6.26)	0.029
Lumbar spine, vertebrae L1 to L4	2.11 (1.20, 3.72)	0.009	1.78 (0.97, 3.26)	0.062	2.66 (1.12, 6.33)	0.027
Total hip	1.98 (1.13, 3.49)	0.018	1.72 (0.94, 3.15)	0.079	2.69 (1.12, 6.46)	0.027
Femoral neck	2.02 (1.14, 3.56)	0.016	1.74 (0.95, 3.19)	0.073	2.73 (1.15, 6.51)	0.023
**WHO osteoporosis**						
Low BMD, lumbar spine	2.17 (1.23, 3.82)	0.008	1.73 (0.94, 3.19)	0.080	2.63 (1.09, 6.32)	0.031
Low BMD, total hip	1.98 (1.12, 3.49)	0.018	1.70 (0.93, 3.12)	0.085	2.75 (1.15, 6.58)	0.023
Low BMD, femoral neck	2.00 (1.13, 3.54)	0.017	1.72 (0.94, 3.16)	0.079	2.74 (1.15, 6.55)	0.024
Low BMD, at least one region	2.08 (1.19, 3.64)	0.010	1.78 (0.97, 3.24)	0.062	2.71 (1.14, 6.46)	0.024

Multivariate models were adjusted for hypertension, hypercholesterolaemia and history of cardiovascular disease. *Analysis of postmenopausal women only. Low BMD is defined as a *T*-score < −1.0 SD. WHO, World Health Organisation.

Within normal BMD, but not within low BMD measurements, there was a tendency towards echolucent carotid plaques to be detected more often in the patients with SLE than in the controls, 34% versus 16%, *P* = 0.067. Prevalence of echogenic plaque did not differ between the study groups at normal and low BMD values.

For bone mass in total body and femoral neck, low BMD and low *T*-scores significantly increased the OR for presence of echolucent plaque versus no plaque in the patients compared with the controls. Thus, for the same total BMD at the hip, the adjusted OR (95% CI) was 2.19 (1.05, 4.55), *P* = 0.036. The point estimates hardly differed when the analysis was performed for total body BMD, low total body BMD and lumbar spine BMD values, though they did not reach statistical significance with adjustments (*P*-values >0.05 and <0.06). In further analysis for presence of echogenic plaque, approximately 4-fold increased ORs were observed for the patients compared with the controls across all BMD measures, thus, ORs (95% CI) were estimated as 3.77 (1.21, 11.71), *P* = 0.022 at the same BMD body total. The results also reflected a higher prevalence of echogenic plaque in the patients than in the controls after adjustment for age, sex, hypertension and hypercholesterolaemia, OR (95% CI) 4.73 (1.21, 18.54), *P* = 0.026, but the estimated group difference was smaller when controlled for history of CVD, 2.39 (0.70, 8.14), *P* = 0.16.

### Factors influencing association between BMD and carotid measurements in patients with SLE

In search of pathomechanisms underlying the association between low BMD and carotid atherosclerosis in patients with SLE, we analysed the effect of potential risk factors for this association, as presented in Table 5. The following factors were found to have a significant effect on detecting with higher cIMT and carotid plaque: age, BMI, ever smoking, systolic blood pressure and a low level of complement C4. The OR of higher cIMT also increased relative to triglycerides and low levels of complement C3 and C4, whereas the OR decreased relative to ever use of antimalarial drugs. On the other hand, additional factors that affected the OR for plaque were blood lipids, diabetes mellitus, history of CVD, glomerular filtration rate (GFR) <60 ml/min/1.73 m^2^, history of nephritis and SLICC.

**Table 5:** **Potential contributors for the relationship between total body bone mineral density (BMD) and carotid measurements in systemic lupus erythematosus (SLE) patients**

**Variable**	**Carotid intima-media thickness mean upper tertile, odds ratio (95% CI)**	***P*** **-value**	**Bilateral plaque, odds ratio (95% CI)**	***P*** **-value**
**Traditional factors**				
Age, per 10-year increase	2.29 (1.73, 2.89)	<0.001	1.68 (1.23, 2.14)	0.003
Female	1.86 (0.46, 7.64)	0.387	1.90 (0.41, 8.87)	0.412
Body mass index, per 1-kg/m^2^ increase	1.18 (1.06, 1.32)	0.002	1.10 (0.99, 1.23)	0.070
Ever smoking, versus never	3.11 (1.29, 7.48)	0.011	2.99 (1.13, 7.93)	0.027
Blood pressure systolic, per 10-mmHg increase	1.43 (1.18, 1.68)	0.001	1.24 (1.01, 1.47)	0.040
Blood pressure diastolic, per 10-mmHg increase	1.18 (0.83, 1.54)	0.312	0.96 (0.61, 1.33)	0.847
Cholesterol total	1.26 (0.88, 1.80)	0.205	1.52 (1.03, 2.23)	0.036
Low-density lipoprotein cholesterol	1.42 (0.89, 2.26)	0.137	2.29 (1.32, 3.99)	0.003
High-density lipoprotein cholesterol	0.61 (0.26, 1.43)	0.254	0.21 (0.07, 0.66)	0.007
Low-density/high-density lipoprotein cholesterol	1.40 (0.86, 2.27)	0.171	3.37 (1.77, 6.42)	<0.001
Triglycerides	2.26 (1.12, 4.57)	0.023	3.14 (1.46, 6.73)	0.003
Diabetes mellitus	-	0.999	6.56 (1.11, 38.65)	0.038
History of cardiovascular disease	1.49 (0.48, 4.63)	0.493	7.44 (2.20, 25.15)	<0.001
**Disease factors**				
High-sensitivity C-reactive protein, per 10 mg/l increase	1.30 (0.61, 2.04)	0.404	0.97 (0.90, 1.51)	0.475
Erythrocyte sedimentation rate, per 10 mm/h increase	1.02 (0.78, 1.27)	0.874	1.17 (0.91, 1.43)	0.206
Low C3 < 0.67 g/l	7.25 (0.86, 60.83)	0.068	1.85 (0.36, 9.41)	0.461
Low C4 < 0.13 g/l	3.04 (1.14, 8.08)	0.026	4.03 (1.25, 13.04)	0.020
Low C1q <70 mg/l	1.04 (0.36, 2.99)	0.941	0.98 (0.31, 3.12)	0.973
Glomerular filtration rate <60 ml/min/1.73 m^2^	1.50 (0.54, 4.12)	0.437	2.77 (1.04, 7.42)	0.042
Disease duration since diagnosis, years, per 10-year increase	1.08 (0.66, 1.52)	0.712	1.00 (0.96, 1.05)	0.869
History of lupus nephritis	1.02 (0.44, 2.39)	0.963	2.47 (1.01, 6.02)	0.047
SLE disease activity index	0.95 (0.87, 1.05)	0.319	0.99 (0.90, 1.09)	0.825
Systemic Lupus International Collaborating Clinics	1.11 (0.91, 1.34)	0.316	1.34 (1.08, 1.66)	0.007
Ever use of antimalarial drugs	0.30 (0.10, 0.92)	0.036	0.89 (0.27, 2.91)	0.850
Ever use of glucocorticoids	2.15 (0.53, 8.76)	0.287	1.25 (0.34, 4.63)	0.741
Total duration of glucocorticoid use, per 10-month increase	1.01 (0.96, 1.06)	0.663	1.00 (0.96, 1.06)	0.682
Total cumulative glucocorticoid dose, per 10-g increase	1.05 (0.83, 1.27)	0.688	1.12 (0.90, 1.35)	0.274
Average daily dose of glucocorticoid	1.03 (0.91, 1.16)	0.694	1.06 (0.93, 1.20)	0.420
Last year cumulative dose of glucocorticoid	0.89 (0.70, 1.13)	0.336	0.81 (0.61, 1.08)	0.148
Last year average daily dose of glucocorticoid	0.96 (0.88, 1.05)	0.336	0.93 (0.84, 1.03)	0.148

Values are OR with 95% CI analysed by multiple logistic regression. cIMT mean upper tertile >0.659 mm.

In multivariate analysis, independent contributors to the relationship between total body BMD and the upper cIMT tertile were age and low C4 level, respective ORs (95% CI) 2.32 (1.73, 2.95) per 10-year increase in age, *P* = <0.001, and 3.21 (1.03, 10.01), *P* = 0.044. Then, age, history of CVD, blood lipids and low C4 were independent contributors to the relationship between BMD and bilateral plaque. The effect of blood lipids, controlled for other factors as above, yielded OR of 2.34 (1.21, 4.49), *P* = 0.011 for low-density lipoprotein (LDL) cholesterol, per each mmol/l, 0.14 (0.04, 0.54), *P* = 0.004 for high-density lipoprotein (HDL) cholesterol, 4.34 (1.90, 9.89), *P* = <0.001 for LDL/HDL, and 3.14 (1.20, 8.21), *P* = 0.020 for triglycerides, but the influence of total cholesterol on this relationship was not statistically significant, OR of 1.43 (0.91, 2.25), *P* = 0.124.

**Figure 3:**
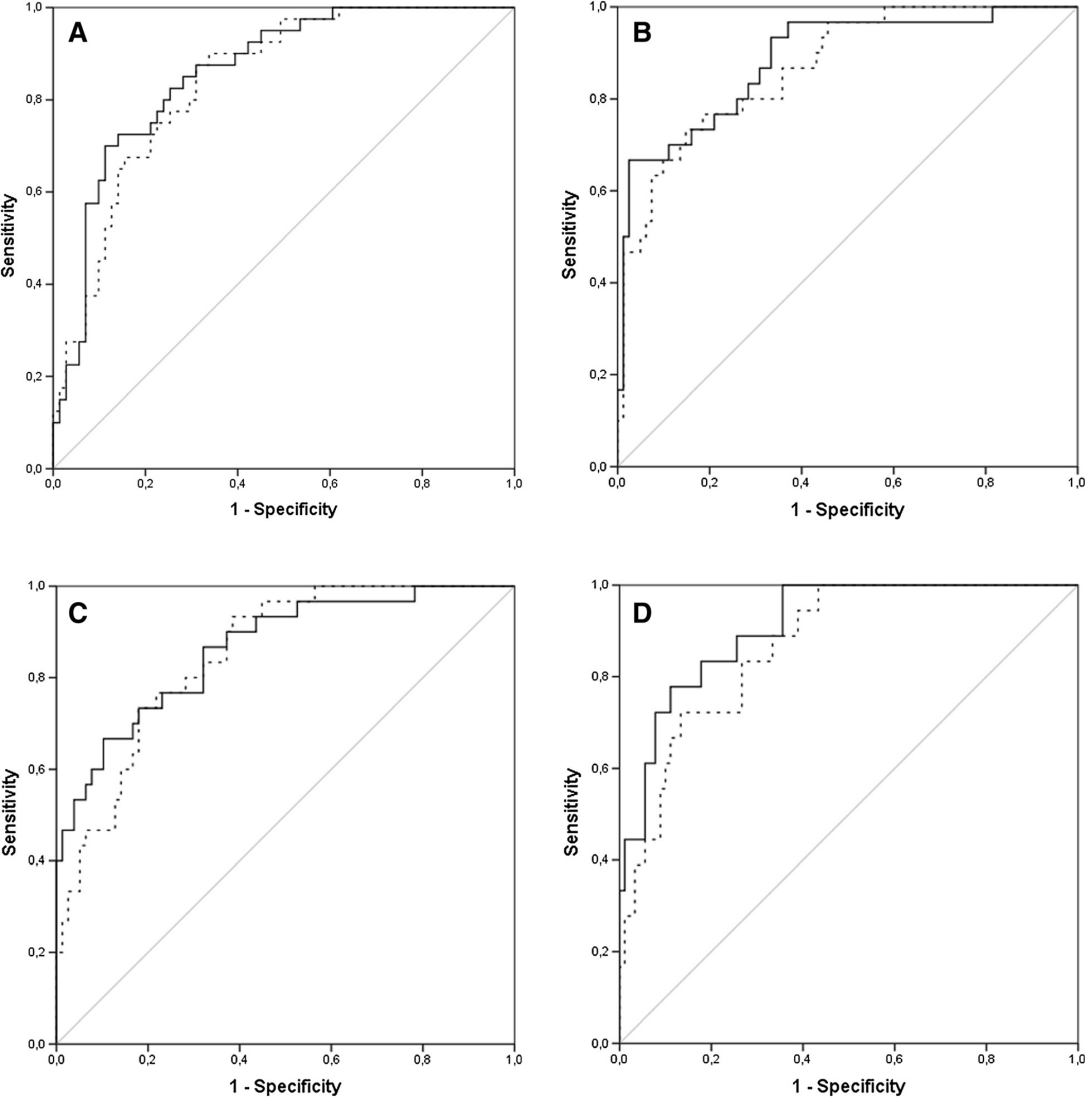
**Receiver operating characteristic (ROC) curves for association between total body bone mineral density (BMD) (A-B) the middle and upper tertiles of total body BMD, ≥1.067 mg/cm** ^**2**^ **(C-D) and the upper mean (>0.659 mm) carotid intima-media thickness (cIMT) (A, C) and bilateral carotid plaque (B, D) in patients with systemic lupus erythematosus (SLE).** The curves are based on multivariate models with traditional risk factors alone (dashed lines) or together with (solid lines) a low level of complement C4 (<0.13 g/l). For total body BMD in association with higher cIMT **(A)** areas under the ROC curves are 0.840 (95% CI 0.768, 0.912, *P* <0.001) and 0.858 (0.789, 0.927, p = <0.001) in corresponding models; and in association with bilateral carotid plaque **(B)** areas are 0.869 (0.797, 0.940, *P* <0.001) and 0.886 (0.813, 0.958, *P* <0.001), respectively. For association between the middle and upper tertiles of total body BMD and upper mean cIMT **(C)**, areas under the ROC curves are 0.850 (0.777, 0.923, *P* <0.001) in analyses incorporating traditional risk factors, and 0.863 (0.786, 0.941, *P* <0.001) in models that also included low C4; respective areas for association with bilateral carotid plaque **(D)** are 0.869 (0.792, 0.946, *P* <0.001) and 0.911 (0.848, 0.974, *P* <0.001).

The receiver operating characteristic (ROC) curve analysis demonstrated that total body BMD explained 65% (95% CI, 54, 75%, *P* = 0.010) of the variance in cIMT measurements and presence of bilateral carotid plaque. The best-fit multivariate model showed that age (OR 1.77, 95% CI 1.17, 2.41, *P* = 0.012), history of CVD (OR 9.15, 95% CI 1.85, 45.40, *P* = 0.007), LDL/HDL (OR 4.34, 95% CI 1.90, 9.89, *P* = <0.001), and low C4 (OR 4.84, 95% CI 1.03, 22.66, *P* = 0.046) were the optimal components that accounted for the association between total BMD and bilateral carotid plaque. The final models explained about 85% (95% CI 77, 96%, *P* = <0.001) of the studied variance in carotid measures and had excellent goodness-of-fit as described in the ROC curves, but the performance of the models improved only modestly with the addition of low C4 to the known traditional risk factors (Figure 3, A-B).

As a difference was observed between patients and controls in the burden of carotid atherosclerosis at (sub)normal BMD values, we next analysed whether disease factors had a stronger impact on the studied association within (sub)normal BMD ranges. The importance of low C4 was found to be enhanced within the second and third tertiles of total BMD (Figure 3, C-D) and particularly pronounced for the association with bilateral carotid plaque. Other disease-specific variables did not contribute independently to the relationship with carotid measures within (sub)normal ranges of total BMD. The findings were consistent with individual BMD measurements (data not shown).

## Discussion

The study is the first to demonstrate an association between BMD and carotid measurements in patients with SLE and age- sex-matched individuals from the general population. Patients with SLE had a higher burden of carotid atherosclerosis than controls, expressed as higher cIMT if they had carotid plaque and found to have low BMD, as well as increased OR for carotid plaque in relation to all BMD measurements, particularly in postmenopausal women. For BMD measures at the hip, we observed an approximately two-fold increase in the OR for the presence of echolucent carotid plaque, and for BMD measures at any sites, more than a four-fold increase in the OR for echogenic plaque in patients compared with controls. Independent contributors to the association between BMD and higher cIMT values in the patients were age and a low C4 level, and for the relationship between BMD and bilateral carotid plaque they were age, history of CVD, blood lipids (LDL and HDL cholesterol, LDL/HDL ratio, triglycerides) and low C4. Total BMD, traditional cardiovascular risk factors and hypocomplementemia C4 together explained about 85 to 90% of the variance in carotid IMT measurements and presence of bilateral carotid plaque. Low C4 seems to be an important disease factor contributing to the relationship between bone status and carotid atherosclerosis in patients with SLE.

The present findings support inverse association between bone mass and carotid measures of atherosclerosis in patients with SLE, in contrast to a recent report [24]. Variation in studied populations, prevalence and definition of outcomes in cross-sectional studies could likely lead to divergent results. Thus, prevalence of osteopenia and osteoporosis in our patients were higher than previously reported in cross-sectional studies investigating bone health in SLE, which could arise due to differences in study populations. Mendoza-Pinto *et al.* found low BMD in 45% of premenopausal SLE patients (mean age 33 years) of mixed-race, with median disease duration of 6 years and lower cumulative exposure to glucocorticoids (mean 20 g) [18]. Lakshminarayanan *et al.*, and Almehed *et al*. reported low BMD in about 60% of patients who were comparable with our study population in terms of demographic and patient characteristics [16,17]. Participants in our study, who were older, had overt atherosclerotic disease, frequent traditional cardiovascular risk factors, and more frequent carotid plaque (45% of patients) than reported by others (11 to 37% of patients) [24,32,33]. Thus, the burden of both low BMD and (sub)clinical atherosclerosis were higher in the present study, which could have influenced the study estimates if patients had not been matched with individuals from the general population.

A number of epidemiological studies have suggested an association between impaired bone metabolism and vascular calcification [34,35]. So far this relationship remains uncertain in autoimmune disease, and has likely been underestimated. Presently, vascular calcification is regarded as an active process, regulated by factors known to be involved in the process of osteogenesis, such as bone morphogenetic protein, osteopontin, the receptor activator of nuclear factor kappa B (RANK) ligand-RANK-osteoprotogerin (OPG) pathway, matric Gla protein and the *Wnt* signalling pathway [14]. Thus, coincidence of vascular calcification with low bone activity could be biologically linked. During the development of vascular calcification, the transition of vascular smooth muscle cells towards an osteoblast-like phenotype and mineralization within these structures is promoted by several players including those related to mineral metabolism [10]. Still, an intriguing question is whether the presence of vascular calcification impacts bone metabolism, thus demonstrating true crosstalk between these tissues. An additional important question is whether or not progression of vascular calcification can be prevented or restricted. Although data are now questioning the safety of supplementation with calcium and vitamin D that may increase vascular calcification and risk of CV events [36], bisphosphonates may act protectively against atherosclerosis [37].

Oestrogen deficiency and premature menopause are considered important risk factors for osteoporosis, and also for atherosclerosis [38]. Recently, increased risk for developing CVD was shown for women with rheumatoid arthritis who had early menopause [39]. Serum lipids, pro-inflammatory cytokines and the RANK system have been suggested as common pathogenic links modulated by oestrogen. Here, in postmenopausal women we found an independent significant association between BMD and carotid plaque. Then, notably, among postmenopausal women, we observed lower hip BMD values in patients compared with controls. Bone microstructure and metabolic activity differ at different sites and bone loss in trabecular bone is considered more pronounced than in mostly cortical bone. In SLE, however, marked loss of cortical area as well as trabecular volume in the femur even without GC treatment, and also a lower hip BMD compared to controls, have been shown [40,41], suggesting deleterious effect of disease per se on bone metabolism. In middle-aged women with SLE, a higher carotid plaque index has been also described in patients in the lower tertiles of total hip BMD, but without a relationship to the lumbar spine bone measures [42]. In elderly women, low BMD in the hip seems to be a marker of atherosclerosis [43]. Thus, the finding of stronger association between low BMD in the hip and echolucent carotid plaque in our study was not unlikely.

To the best of our knowledge, no previous study has examined whether carotid plaque morphology, in terms of echogenicity, relates to BMD in SLE. In the general population, it has been reported that the prevalence of echogenic plaque, having a higher content of calcified material and fibrous tissue, was significantly related to low BMD, but no association was found between BMD and echolucent plaque, having a higher content of lipid, necrotic debris and/or haemorrhage [44]. Here, we observed a two-fold increased OR for echolucent plaque in the patients than in the controls at the same hip BMD values, and a four-fold increase for echogenic plaque with adjustment for age, sex, hypertension and hypercholesterolaemia. The estimated group difference for detection with echogenic plaque was smaller when controlling for history of CVD, but overt atherosclerotic disease is in the causal pathway with carotid plaque, and to which degree adjustments should be made when assessing the strength of association between osteoporosis and carotid measurements is unclear. Notably, the prevalence of echolucent carotid plaque was numerically higher in the patients than in the controls within normal BMD values, and echogenic plaques were found at lower cIMT values in patients than in the controls with that type of plaque within normal BMD. Further, plaque of both types of echogenicity were detected at numerically lower cIMT values in the patients compared with the controls within normal BMD. In line with our observation, Frerix *et al.* frequently found atherosclerotic plaque lesions in the absence of intima-media thickening in both SLE and systemic sclerosis patients [45]. Altogether, our results suggest that patients with SLE may already have premature atherosclerosis within BMD ranges of normal bone mass.

We observed that LDL cholesterol, LDL/HDL ratio and triglycerides were among the factors independently inversely related to the association between BMD and carotid measurements in patients, whereas HDL cholesterol showed a protective effect. Several studies *in vitro* have previously suggested that abnormal lipid profile may be the key factor responsible for the inverse association between BMD and atherosclerosis, as lipids modulate bone remodeling and the atherosclerotic process in opposite directions [46,47]. This suggestion however has not been verified in human studies [48,49], and in a study of elderly women none of the lipids contributed independently to the variation in BMD [50]. The role of lipids in regulation of bone mass in SLE is unclear. Taking into account the evidence linking inflammatory altered lipid profile to premature atherosclerosis in SLE, it is reasonable to hypothesize that lipids may be in the pathogenic pathway and relate to bone loss too.

The deleterious effect of the SLE-disease per se on bone metabolism is supported by clinical studies, which suggest there is lower BMD in SLE patients even without prior GC exposure, compared with healthy controls [51]. It is also well-established that a high cumulative inflammatory burden in SLE patients is associated with reduced BMD and presence of atherosclerosis [19,23]. Our present study further addressed this issue and the results of univariate analysis suggest that the low level of complement C3 and C4, low GFR, history of nephritis, damage-index SLICC, and ever use of antimalarial drugs could be of importance in the relationship between disease and bone health, and arterial vessel health. Of these, hypocomplementaemia C4 was an independent factor for the relationship, particularly within (sub)normal ranges of total BMD. It has been implicated that many pathogenetic mechanisms behave paradoxically in inflammatory condition, compared with general population. Thus, substantial positive correlation of higher complement factors C3 and C4 with cardiovascular risk factors and risk of CVD have been reported in population-based studies [52]. Whether complement function regulates bone turnover, for example, indirectly through effects on the immune system, is unknown. In the present study, multivariate analysis controlling for BMD, age, sex, hypertension, hypercholesterolaemia, history of CVD, lipids and low C4, together explained about 85% of the variance in carotid measurements. Although the performance of adjusted models improved only modestly with addition of low C4, traditional and inflammatory dependent risk factors together may more accurately define the processes that lead to the development of bone loss and atherosclerosis.

The SLE patients in our study had mild disease activity and low severity and were on long-term, low-dose GC therapy. Therefore one cannot infer a similar effect size in other patient populations. Furthermore, the SLE patients were given supplements of calcium with vitamin D and treated with bisphosphonates, which might have played a role in minimizing the study estimates.

## Conclusion

This study demonstrates associations between BMD and carotid measures in patients with SLE and population-based matched controls and suggests a higher burden of carotid atherosclerosis at the same bone mass in patients than in controls. Longitudinal studies are needed to prove the pathophysiological relationship. The message for clinical practice is that diagnosis and treatment of bone loss in patients with SLE should include consideration of cardiovascular risk assessment to improve cardiovascular outcomes; conversely, detection of (sub)clinical atherosclerosis should prompt consideration of densitometry to diagnose and prevent complications of osteoporosis.

## Abbreviations

ACR: American College of Rheumatology
BMD: bone mineral density
BMI: body mass index
CCA: common carotid artery
cIMT: carotid intima-media thickness
CVD: cardiovascular disease
DMARD: disease-modifying anti-rheumatic drug
DXA: dual-energy x-ray absorptiometry
ESR: erythrocyte sedimentation rate
GC: glucocorticoids
GFR: glomerular filtration rate
HDL: high-density lipoprotein
HRT: hormone replacement therapy
hsCRP: high-sensitivity C-reactive protein
LDL: low-density lipoprotein
OPG: osteoprotegerin
OR: odds ratio
RANK: receptor activator of nuclear factor kappa B
ROC: receiver-operating characteristic
SLE: systemic lupus erythematosus
SLEDAI: systemic lupus erythematosus disease activity index
SLEVIC: systemic lupus erythematosus vascular impact cohort study
SLICC: Systemic Lupus International Collaborating Clinics
WHO: World Health Organisation
